# FluxCTTX: A LIMS-based tool for management and analysis of cytotoxicity assays data

**DOI:** 10.1186/1471-2105-16-S19-S8

**Published:** 2015-12-16

**Authors:** Alessandra C Faria-Campos, Luciene B Balottin, Gianlucca Zuin, Vinicius Garcia, Paulo HS Batista, José M Granjeiro, Sérgio VA Campos

**Affiliations:** 1 Department of Computer Science, Universidade Federal de Minas Gerais, Antônio Carlos Avenue, 6627, Pampulha, Belo Horizonte, MG, 30123-970, Brazil; 2 INMETRO, Nsa.Sra.das Gra¸cas Avenue, 50, Xérem-Duque de Caxias, RJ, 25250-020, Brazil

**Keywords:** Laboratory Information Management Systems, Workflow, Cytotoxicity Tests, OECD129, Good Laboratory Practices, Interlaboratory comparison

## Abstract

**Background:**

Cytotoxicity assays have been used by researchers to screen for cytotoxicity in compound libraries. Researchers can either look for cytotoxic compounds or screen "hits" from initial high-throughput drug screens for unwanted cytotoxic effects before investing in their development as a pharmaceutical. These assays may be used as an alternative to animal experimentation and are becoming increasingly important in modern laboratories. However, the execution of these assays in large scale and different laboratories requires, among other things, the management of protocols, reagents, cell lines used as well as the data produced, which can be a challenge. The management of all this information is greatly improved by the utilization of computational tools to save time and guarantee quality. However, a tool that performs this task designed specifically for cytotoxicity assays is not yet available.

**Results:**

In this work, we have used a workflow based LIMS -- the Flux system -- and the Together Workflow Editor as a framework to develop FluxCTTX, a tool for management of data from cytotoxicity assays performed at different laboratories. The main work is the development of a workflow, which represents all stages of the assay and has been developed and uploaded in Flux. This workflow models the activities of cytotoxicity assays performed as described in the OECD 129 Guidance Document.

**Conclusions:**

FluxCTTX presents a solution for the management of the data produced by cytotoxicity assays performed at Interlaboratory comparisons. Its adoption will contribute to guarantee the quality of activities in the process of cytotoxicity tests and enforce the use of Good Laboratory Practices (GLP). Furthermore, the workflow developed is complete and can be adapted to other contexts and different tests for management of other types of data.

## Background

Cytotoxicity is the quality of being toxic to cells. Examples of toxic agents are some types of venom (e.g. from the puff adder or brown recluse spider). Treating cells with the cytotoxic compound can result in a variety of cell fates: The cells may undergo necrosis, stop actively growing and dividing or activate a genetic program of controlled cell death (apoptosis). Cytotoxicity assays are the tests used by researchers to screen for cytotoxicity in compound libraries. Researchers can either look for cytotoxic compounds, if they are interested in developing a therapeutic that targets rapidly dividing cells or they can screen "hits" from initial high-throughput drug screens for unwanted cytotoxic effects before investing in their development as a pharmaceutical.

Cytotoxicity tests may be used as a substitute to *in vivo *tests that use animals. The concept of using *in vitro *cytotoxicity data to determine the starting doses for rodent acute oral toxicity tests was discussed and evaluated at an International Workshop on *In Vitro *Methods for Assessing Acute Systemic Toxicity convened in 2000 [1]. The approach involves using an IC50 value from an *in vitro *basal cytotoxicity test with the Registry of Cytotoxicity (RC) regression to predict an LD50 value for use as a starting dose for the Acute Toxic Class (ATC) method or the Up-and-Down Procedure (UDP) test method [2]. Simulations showed that using *in vitro *cytotoxicity assays to estimate an LD50 to use as a starting dose in the UDP could potentially reduce animal use by 25-40%. Additionally, several tests have currently demonstrated the efficiency and effectiveness of alternative methods testing to reduce, refine, and/or replace the use of animals in testing [1-5].

However, to rely on these assays it is very important that quality is assured for all tests through a well-designed Quality Management System (QMS) which includes tools to register and verify the information regarding the tests. For research laboratories two important QMS are the management systems from the International Organization for Standardization (ISO) and the Good Laboratory Practices (GLP). Good Laboratory Practices are defined by the Organization for Economic Cooperation and Development (OECD) as "a quality system concerned with the organizational process and the conditions under which non-clinical health and environmental safety studies are planned, performed, monitored, recorded, archived and reported" [6,7]. The purpose of the GLP principles is to promote the development of quality test data and provide a tool to ensure a sound approach to the management of laboratory studies, including conduct, reporting and archiving. The GLP principles may be considered as a set of standards for ensuring the quality, reliability and integrity of studies, the reporting of verifiable conclusions and the traceability of data. The ISO management systems provide a model to follow when setting up and operating a laboratory according to a set of standards internationally recognized. They are the result of international, expert consensus and therefore offer the benefit of global management experience and good practice [8].

The recognition of compliance to the quality management principles by laboratories can be complex. This process can be slow and costly, potentially requiring changes in laboratory routine. Recognition and maintenance of compliance to these principles is also difficult because it requires that the laboratory maintains a continuing routine of activities that include staff training, maintenance of facilities and equipment, monitoring the quality of the documents and lab records, calendar inspections and periodic reporting. Manual capture, calculation and verification of raw data result in a tremendous drain on human resources while also jeopardizing the integrity of the information. The administration of paper records is particularly inefficient and expensive and data cannot be easily integrated with other technologies. As a result, complying with the strict principles of quality systems can be a very time consuming and expensive process. Thus, the use of computer systems capable of addressing the complexity of the regulations, ensuring compliance with current best practice and satisfying the concerns and expectations of the regulators is pressing. The main tools that can assist in this process are the Laboratory Information Management Systems **(LIMS)**, which allow the recording of lab activities in a complete and easy way. All the data are recorded in databases, the relationship between the experimental steps is logged, allowing the traceability of samples and its chain of custody, reagents and results [9,10]. An extensive list of LIMS can be found nowadays and several LIMS are currently available as academic, proprietary and open source solutions. Some examples of these include SQL LIMS [11], Lab-Soft [12], LabWare [13], FreeLIMS [14], Biotracker [15], Watson [16] and the systems developed by Hendrick [17], Quo [18], Tharayil [19] and Sanchez [20].

One of the main difficulties to implement a LIMS is the fact that each laboratory has a different routine of experimentation that changes over time. Therefore some LIMS propose a customization to adequate the LIMS general structure to the needs of the laboratory [15,16]. However, this customization can be time consuming and may sometimes not address all the needs of a specific laboratory, such as those performing the cytotoxicity tests. Moreover, although a significant number of LIMS are available today, none of them addresses specifically the information on cytotoxicity assays and therefore more general ones are used to store these data, but typically the analysis is performed manually through independent analysis tools [21]. Other works mention cytotoxicity assays being managed by in-house LIMS, being therefore unavailable for comparison [22]. There are LIMS that provide some functionality for cytotoxicity data, such as the one provided by *Thermo Scientific*, which provide an image analysis application [23]. In these cases, however, the LIMS is not used for a complete cytotoxicity analysis, only for part of it.

This work proposes the use of a workflow based LIMS -- the Flux system -- and the Together Workflow Editor as a framework to develop FluxCTTX, a tool for management of data from cytotoxicity assays performed at different laboratories. The main work is the development of a workflow, which represents all stages of the assay and has been developed and uploaded in Flux. This workflow models the activities of cytotoxicity assays performed as described in the OECD 129 Guidance Document [6], and guarantees that experiments performed adhere to the GLP principles.

## Methods

The design and construction of FluxCTTX involved the use of a LIMS (with an associated database server) as an engine and a workflow editor to construct the forms for the interface.

### The Flux System

Flux is a LIMS that has been constructed using *Java *technology and uses MySQL as a database server and Apache Tomcat as a Web server. The system has a web interface which is platform-independent and can be accessed using the main browsers (Internet Explorer, Google Chrome, Mozilla Firefox and Safari). Workflow files are uploaded in the system through its web interface. The activities created in the workflow construction are then interpreted by the system as links.

The workflow **CTTX **has been constructed using the Together Workflow Editor (TWE) [24] in the XML Process Definition Language (XPDL) file format. The Flux system is able to represent different workflows modeled in TWE in the XPDL format according to the needs of each experimental design. In Flux the protocols are defined as processes that are composed of steps, referred to as activities. An activity represents events of a process and, as such, has transitions, actors and rules. This information is represented as attributes in the workflow definition. Therefore, the user can define the characteristics of the attributes of each activity, such as its types, the range of values that each attribute can assume, its formats or even define auto-calculated attributes derived from other attributes. In TWE, inputs and outputs of each activity are also defined, including the relation of these with the experiments. During workflow definition it is also possible to assign to each activity a documentation that contains standard protocols, instruments calibration, procedures and records associated with the activities.

**Figure 1:**
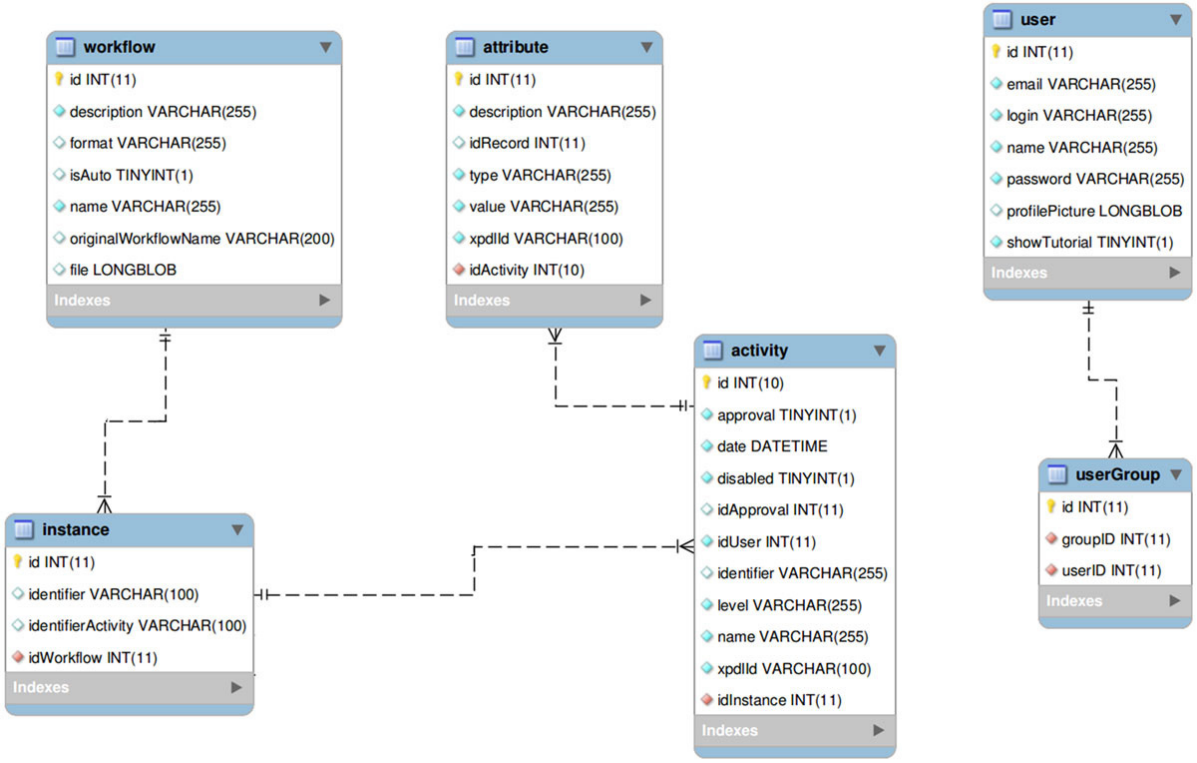
**FluxCTTX database scheme**.

All the information regarding the activities and attributes is stored in a entity-relationship database through an activities table that stores all the data, using metadata to indicate, whenever necessary, to which experiment or activity each data belongs to. A simplified entity-relationship diagram for this is showed in Figure 1.

### The cytotoxicity assay modeled in FluxCTTX

The cytotoxicity assay modeled in FlucCTTX was the Neutral Red Uptake (NRU) *in vitro *basal cytotoxicity assay procedure described by the *OECD 129 Guidance Document on Using Cytotoxicity Tests for Estimate Starting doses for Acute Oral Systemic Toxicity Tests *[6]. The test is based on the ability of viable cells to incorporate and bind neutral red (NR), a supravital dye. Toxicants can alter cell surface or lysosomal membranes that causes cell death and or cell growth inhibition, which decreases the amount of NR retained by the culture. The concentration of NR dye desorbed from the cultured cells is directly proportional to the number of living cells, so cytotoxicity is expressed as a concentration-dependent reduction of the uptake of NR after chemical exposure. The NRU assay uses a 96-well plate format that is reproduced in the system. The light absorption (Optical density - OD) at 540 nm within 60 minutes of adding NR is measured using microtiter plate reader (spectrophotometer) using blank as reference. Values of OD are registered in the system and the averages for OD of the blank are calculated. Two controls are present in the test: (i) a Positive Control(PC) where the test is performed using Sodium dodecyl sulphate (SDS) as test substance to obtain a complete dose-response curve and (ii) a Vehicle Control(VC), that consists of routine culture medium when the test substances are dissolved in culture medium. Cell viability is calculated automatically by the system for each test well as percent of the mean VC at OD540. The IC50 of the test substance is automatically calculated by the system using the statistical package R, using a Hill function analysis of the replicate cell viability data for each concentration.

### The Construction of the CTTX workflow

The development of FluxCTTX consisted of the construction of the CTTX workflow and its upload to the LIMS Flux. The instructions for the cytotoxicity assay of the OECD 129 Guidance Document have been used for the workflow construction. Information regarding the set up of the assay as well as its results have been described in the systems as attributes that are detailed according to the information stored. There are several different types of attributes, each fulfilling a specific need in storing and managing the data. In this workflow each activity corresponds to an experimental step, and its attributes identify the types of information that are generated in this experimental step. The sequence of activities then contain all the steps of assays performed. The attributes in each activity store the specific information of that activity. As a consequence, the workflow has the complete description of the data being managed, all the steps and for each step all the required data. Because of this, the FluxCTTX system has been fully developed by specifying the workflow, without need to change the code of the LIMS. Other workflows, or adaptations to this workflow can be developed by changing the workflow files in the editor, without changing the Flux system. The information managed by the FluxCTTX system is completely contained in its workflow, the Flux system is the engine that drives the workflow. It is used to exhibit the information contained in the workflow, that is self-contained in the same way as a spreadsheet or text file. The file contains the data and all of the formulas that are required to understand it and FluxCTTX is an engine to understand and process these data. Moreover, for those attributes that represent results of calculations, the system performs those, automatically preventing human errors.

## Results

**Figure 2:**
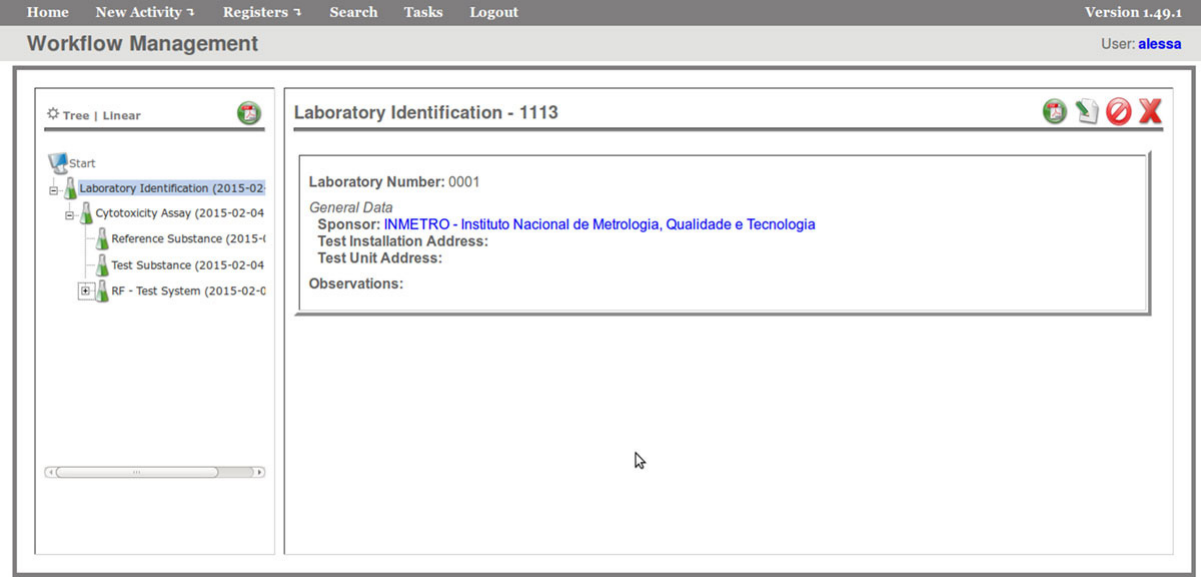
**FluxCTTX interface showing the main activities of the CTTX workflow**.

**Figure 3:**
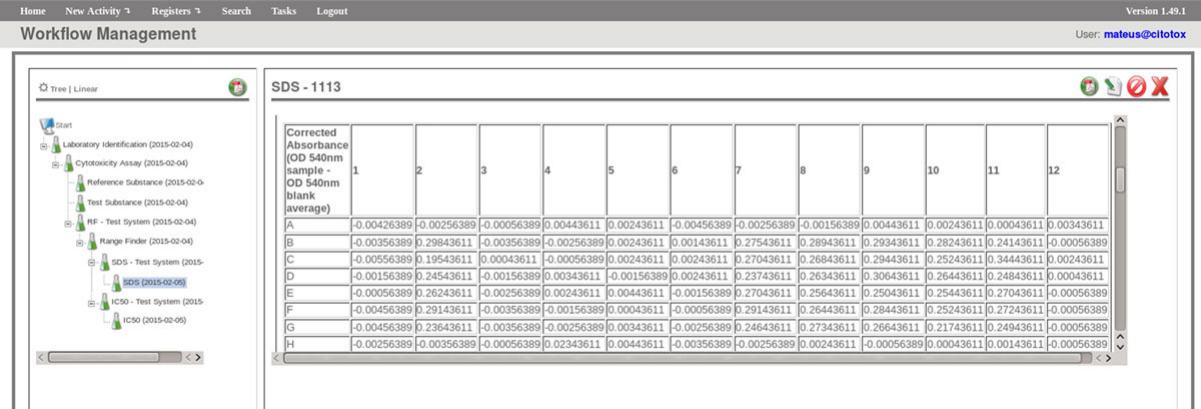
**FluxCTTX interface showing form for corrected optical density (light absorption) calculations**.

**Figure 4:**
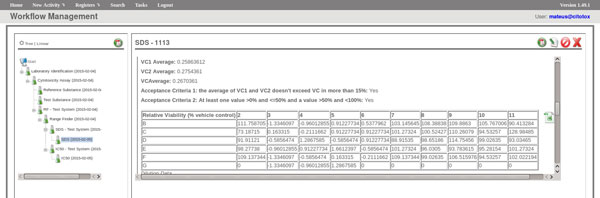
**FluxCTTX interface showing form for cell viability calculations**.

**Figure 5:**
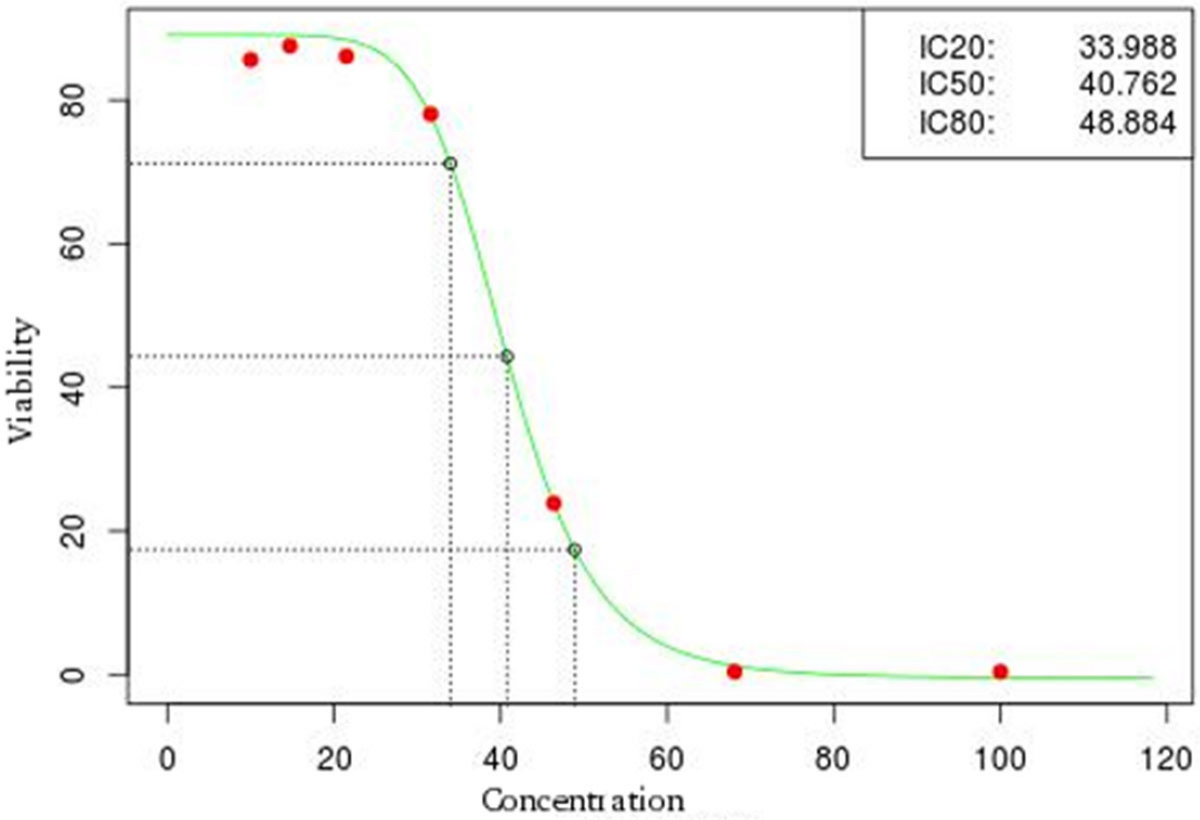
**FluxCTTX interface showing a graphic for IC50 calculation constructed automatically by the system FluxCTTX using regression calculated data without users assistance**.

The FluxCTTX workflow has been developed using TWE and has 4 main activities, representing the steps of the OECD129 protocol and 3 child activities (Figure 2). The first activity -- *Laboratory identification *--identifies the laboratory, the test performed, reagents used and team involved in the procedure. The following activities keep the information regarding the range finder for the test substance, the test itself (IC50) and the positive control. These forms have the information on samples used and the values of optical density (OD) at 540 nm for the tests, including the corrected OD, calculated by the system subtracting the OD values obtained for the blank (average) from those obtained for the test substance (Figure 3). The workflow also shows the values for cell viability calculated automatically by the system (Figure 4). Moreover, the values of IC50 are also calculated automatically by the system using the R package and the graphic for the calculations is exhibited (Figure 5).

The first test of FluxCTTX has been made by inviting 5 different laboratories in Brazilian Institutions that perform cytotoxicity assays to participate in a Interlaboratory comparison where the information regarding the assays was uploaded in the FluxCTTX system. The users in these laboratories inserted the data of the experiments performed and checked the calculations performed by the system with those performed manually using the Microsoft Excel Spreadsheet. The results matched. A total of 24 assays have been performed and the data from those uploaded in the FluxCTTX system. The results are summarized in Table 1.

**Table 1:** Summary of FluxCTTX experiments.

Lab #	Number. of exp.	Average IC50	STDEV	results of CTTX assays
CI001	4	24.21	0.80	unsatisfactory
CI002	3	0	-	unsatisfactory
CI003	15	44.12	6.08	satisfactory
CI004	2	73.3	1.09	unsatisfactory
		29.03	40.1	unsatisfactory
CI005	0	0	-	unsatisfactory

Lab 2 completed 3 experiments, but was unable to compute an IC50. Lab 4 reported 2 experiments, but did not use FluxCTTX.

The director of the study had access to all the data inserted in the system and was able to compare the data inserted by the five different laboratories in a very efficient and precise way.

## Discussion

FluxCTTX has been used in a Interlaboratory comparison by five different laboratories located in three different Brazilian states. Each laboratory has different coordinators and use different experimentation techniques. Because of this diversity, in previous laboratory comparisons, each has performed the experiments slightly different than the others, and comparing the results has been difficult. In addition to that, some laboratories have not previously complied with the GLP principles. Due to the lack of standardization in laboratory procedures it became difficult not only to compare results, but also to identify the reasons why results vary among these. IC50 values have been computed showing that FluxCTTX can accurately compute automatically the required values directly from the experimental data. Previously, in these labs the experimental data had to be manually input in a separate statistical analysis tool, potentially leading to errors. Moreover, different tools calculate the IC50 slightly differently, leading to results that could not be directly compared. It is important to notice that because there are no other cytotoxicity LIMS available to the laboratories, a direct comparison between FluxCTTX and another CTTX LIMS is not possible. In some cases the LIMS is not available to external users [22]. In other cases, the systems available only automate part of the analysis [21,23], and a manual component equivalent to the manual analysis mentioned above is performed.

All results of the comparison have been inserted in the system, except for one. One laboratory did not follow the OECD 129 standard protocol for the experiments and reported their results without using the FluxCTTX system. The IC50 values reported by this group have had the largest variance of all experiments, indicating that adherence to the standard protocol increases the accuracy of the experiments. This is not a definite indication, since the number of experiments was too small, but it is a clear one, since the only lab that did not follow the protocol was the only one that was unable to analyze their results in the system, and the one with the less accurate results. Notice that without an integrated method of managing the data and reporting the results, the coordinator of the comparison would have to rely only on reports sent by the laboratories. In most cases the reports would have been different, and a comparison would be difficult. In particular, it would be very difficult to identify that a lab did not follow the standard protocol, and the relation between adherence to protocol and more accurate results might not have been found.

Of the experiments uploaded in the system, not all have been considered satisfactory. FluxCTTX managed, however, to store data from all experiments and helped to identify the better ones by computing the IC50 values. It is important not only to identify the best experiments, but also to store the unsatisfactory ones for comparison purposes. We are currently sifting through the data stored to identify the reasons for the success or failure of experiments. If common patterns exist that indicate success or failure of experiments, this information can be used to suggest actions in future experiments to increase the likelihood of a successful experiment.

## Conclusion

In this work we present the development of the FluxCTTX system, a LIMS designed to manage and analyze cytotoxicity assays performed in accordance to GLP principles and the protocols of the OECD 129 Guidance Document. FluxCTTX manages experimental data and analyzes them, computing IC50 values and suggesting if the experiments are satisfactory or not. FluxCTTX has been successfully used in a Interlaboratory comparison with five different laboratories, making it simpler to follow the standard protocol, to report results, and to compare these results. Future comparisons including a larger number of laboratories are planned, which should provide more data to be analyzed not only to assess the effectiveness of FluxCTTX, but also to help identifying the best experimental procedures.

The FluxCTTX system will be available at http://syrah.luar.dcc.ufmg.br/fluxcttx[25].

## List of Abbreviations

• CTTX - Cytotoxicity

• GLP - Good Laboratory Practice

• LIMS - Laboratory Information Management System

• OECD - Organization for Economic Cooperation and Development

• SDS - Sodium Dodecyl Sulphate

• TWE - Together Workflow Editor

• XPDL - XML Process Definition Language

## Competing interests

The authors declare that they have no competing interests.

## Authors' contributions

ACFC, GZ and VG constructed and tested the CTTX workflow XPDL files. PHB, VG and SVAC developed and implemented the LIMS Flux. LBB and JMG inserted the data on FluxCTTX, coordinated and performed the Interlaboratory comparison. All authors have helped in preparing the manuscript.

## Declarations

Publication charges for this article have been funded by INMETRO and Computer Science Department at Federal University of Minas Gerais.

This article has been published as part of *BMC Bioinformatics *Volume 16 Supplement 19, 2015: Brazilian Symposium on Bioinformatics 2014. The full contents of the supplement are available online at http://www.biomedcentral.com/bmcbioinformatics/supplements/16/S19
